# Research Note: Changes in eggshell quality and microstructure related to hen age during a production cycle

**DOI:** 10.1016/j.psj.2021.101287

**Published:** 2021-05-27

**Authors:** Cristina Benavides-Reyes, Elisa Folegatti, Nazaret Dominguez-Gasca, Gilberto Litta, Estefania Sanchez-Rodriguez, Alejandro B. Rodriguez-Navarro, Murtala Umar Faruk

**Affiliations:** ⁎ Department of Mineralogy and Petrology, University of Granada, 18002 Granada, Spain; † DSM Nutritional Products, Via G. di Vittorio 20090 Segrate, Milan, Italy; ‡ DSM Nutritional Products, Animal Nutrition and Health, Wurmisweg 576, 4303 Kaiseraugst, Switzerland; § DSM Nutritional Products, 1, Boulevard d'Alsace, 68128, Village-Neuf, France

**Keywords:** eggshell quality, microstructure, age, hen, production cycle

## Abstract

We have studied in detail the changes that occur in eggshell structure and composition during a production cycle in order to better understand the deterioration of eggshell quality with hen age (at 33, 45, and 67 wk). To study changes in eggshell ultrastructure and microstructure characteristics (mammillary density, palisade layer thickness, size, and orientation of calcite crystals) and the cuticle composition, we used complementary analytical techniques such optical and electron microscopy, X-ray diffraction, and infrared spectrometry. The marked decrease in eggshell breaking strength from 5.8 Kg at 33 wk to 4.4 Kg at 67 wk (25% reduction) could not be solely explained by the modest reduction in eggshell thickness (6–10% reduction) and seems to be associate to abrupt changes in eggshell ultra- and microstructure characteristics (i.e., decreased mammillary density; increased size of crystal units), occurring in older hens. Particularly, the decrease in mammillary density reduces the attachment points of the eggshell mineral to the membranes and therefore should negatively impact eggshell mechanical properties. Also, the observed increase in the calcite crystal size making the shell could also reduce the cohesion of crystals and eggshell resistance against impacts. Additionally, there was a decrease in the amount of cuticle and internal egg quality parameters (egg albumen height) with hen age that could have a negative impact in egg safety and quality.

## INTRODUCTION

**Figure 1:**
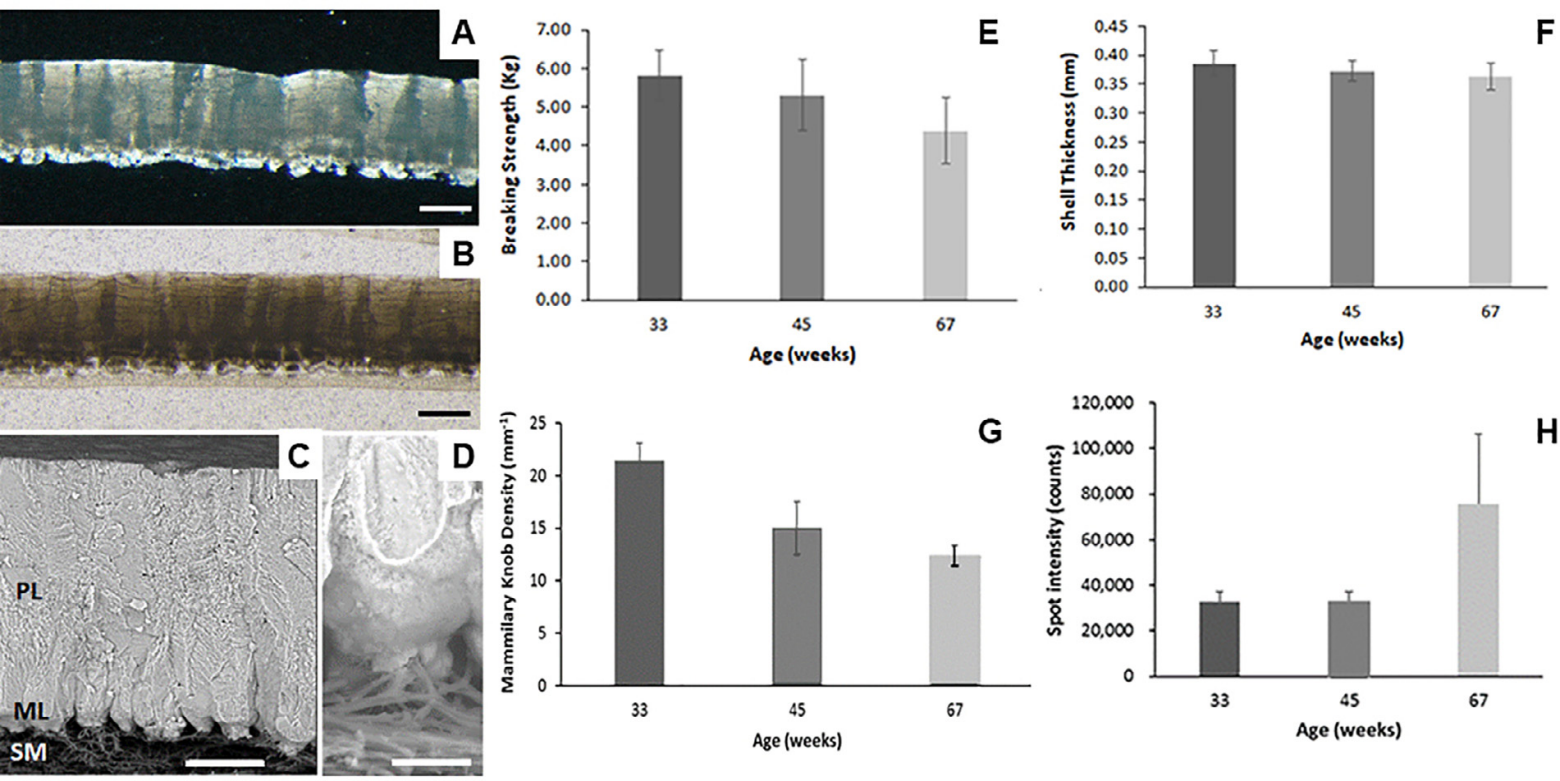
Eggshell ultra- and microstructure analysis and mechanical properties. Optical microscopy images of an eggshell cross-section as viewed under cross-polarized (A) showing columnar calcite crystals with varying degrees of light extinction due to differences in their crystallographic orientation or parallel light (B) illumination showing that the mineral part has a significant amount of occluded organic matter with a greater concentration in the mammillary layer. (C, D) Scanning electron microscopy images (BSE mode) display the general eggshell structure at higher magnification showing that the mineral part is dense and that mammillary cores are firmly attached to the outer eggshell membrane fibers. Scale bars are: (A, B) 200 µm. (C) 100 µm. (D) 50 µm. (E) Eggshell breaking strength. (F) Eggshell thickness at the egg equator. (G) Mammillary density and (H) Average intensity of diffraction spots. Abbreviations: ML, mammillary layer; PL, palisade layer; SM, shell membranes.

Eggs are a complete and inexpensive source of proteins, fat-soluble vitamins (A, D, and E), and water-soluble vitamins (vitamin B12, riboflavin, and folate) as well as several micronutrients (e.g., iodine, iron, phosphorus, and selenium) (Réhault-Godbert et al., 2019). The egg content is protected against mechanical impacts, microbial contamination and dehydration, by the eggshell, a thin mineral layer, which is of upmost importance to preserve egg nutritional qualities and food safety (Hincke et al., 2012). The eggshell mineral part is made of calcite columnar crystal units (palisades), about 70 to 80 µm wide, extending across its thickness (about 330 µm in chickens; Figure 1). Calcite columnar units radiate from the mammillary cores which are anchored in the eggshell membranes on specific organic rich sites (mammillary knobs) that act as nucleation centers of calcite crystals. The mineral part has a significant amount of occluded organic matter that reinforced the otherwise brittle calcite crystals, making the eggshell a very tough material given its small thickness. On the other hand, the eggshell is coated by a very thin organic layer (the cuticle) that plugs the pores and prevents bacterial ingress through the shell (Bain et al., 2013).

The eggshell characteristics related to quality are greatly influenced by a wide array of factors including genetics, age, nutrition, and environment (housing, lightning program) (Dunn et al., 2009; Nys, 2017). Despite genetic selection and improved animal husbandry practices, eggshell quality deteriorates with hen age during the production cycle (Rodriguez-Navarro et al., 2002; Roberts et al., 2013; Bain et al., 2016). In the same way, egg internal quality (i.e., egg albumen height) also deteriorates with hen age. Egg breakage is and always has been an economical drain to the egg industry. Cracked and damaged eggs accounts for about 8% of total egg production that need to be downgraded, thus causing substantial economic loss to egg producer. This percentage can rise to 20 to 30% at the end of the laying cycle (65–70 wk) being one of the main reasons to limit the production to that age. Poor eggshell quality is also a major concern for food safety as eggs with a damaged eggshell are more easily contaminated with bacteria (Bain et al., 2013). The cuticle also reduces moisture and CO_2_ loss through the shell, which slows down the natural decline of egg internal quality during storage.

The objective of this experiment is to study the changes that occur in eggshell structure and composition during a production cycle in order to better understand the deterioration of eggshell quality with hen age. The study collected and evaluated eggs from a flock of hens at 33, 45, and 67 wk of age using complementary analytical techniques such optical and electron microscopy, X-ray diffraction, and infrared spectrometry to study changes with hen age of eggshell ultrastructure and microstructure characteristics (mammillary density, palisade layer thickness, size, and orientation of calcite crystals) and the cuticle composition. All these parameters have an important contribution to eggshell physical properties (i.e., eggshell strength, shell permeability) and should be considered for a complete assessment of eggshell quality. Additionally, changes with age of most relevant egg internal quality parameters (i.e., albumen height, yolk color) were also evaluated.

## MATERIALS AND METHODS

### Animals and Housing

The analyzed eggs were collected from a flock of 3,150, 18-wk-old, LSL-hens, placed into a layer barn (7.4 hens/m^2^) on deep-litter (1.2 kg/m^2^ were used to collect the analyzed eggs. Lighting program was a 15L:9D. Mash feed was provided ad libitum. The diet was a wheat-soybean meal-based diet and formulated for 2 different feeding phases (phase 1: Ca 36 g/Kg, from wk 21 to 44; and phase 2: Ca 41 g/Kg, wk 45 to 68 of hens' age).

### Egg Physical Properties

Basic egg properties (egg weight, eggshell thickness, egg albumen height, and yolk index [**YI**]) were determined for each egg, using the NABEL DET6000 egg quality analyzer (NABEL Co. Ltd. Kyoto 601-8444 Japan). Additionally, eggshell thickness was measured with a micrometer in each egg in 3 points in the egg equator.

### Microscopy

For microstructure analysis of eggshell samples using optical microscopy, thin sections of a piece of shell from the egg equator were prepared from 14 samples of each hen age group. Samples were embedded in epoxy resin (Epothin, Buehler, Germany), and studied using a Nikon LSZ 1000 microscope using polarized light. The outer surface and ultrastructure in the cross-section of eggshell samples were also examined by electron microscopy. The eggshells were coated with carbon (Hitachi UHS evaporator, Hitachi Ltd., Tokio, Japan) and observed with an SEM (Hitachi S-510, Hitachi Ltd.) using backscattering electron mode (**BSE**) at an accelerating voltage of 20 keV.

### X-ray Diffraction

The size and orientation of crystals in the eggshell was analyzed using an X-ray single crystal diffractometer equipped with a CCD area detector (D8 SMART APEX; Bruker, Germany) using a Mo X-ray tube operated at 50 kV and 35 mA and a 0.5 mm collimator. A piece of shell (1 × 1 cm) cut from the egg equator was measured by transmission with a 30 s exposure time. From the collected pattern, the number and intensity of spots and crystal orientation (I110/I104 ratio) were determined using XRD2DScan 7.0 software (PANalytical, The Netherlands).

### Chemical Analysis

The amount and chemical composition of the cuticle in each egg was analyzed by infrared spectroscopy. Briefly, the outer surface of an intact fragment of eggshell was pressed against the ATR diamond crystal window (ATR Pro ONE) and the IR spectra recorded at a 2 cm^−1^ resolution using a FTIR spectrometer (model 6200, JASCO, Tokyo, Japan). Additionally, the percentage of water, organic matter, and mineral content were determined by thermogravimetry. Powdered eggshell samples were treated at different temperatures (200, 400, and 600 C) for 1 h and weighed to determine the weight percentage of each component.

### Statistical Analysis

Basic descriptive statistics were used to characterize basic egg properties. The data were expressed by (mean± standard deviation) and were analyzed for statistical significance using one-way analysis of variance (**ANOVA**), followed by Tukey post-hoc test. Pearson's correlation analysis and multivariate linear regression models were used to study the relationships between the different eggshell characteristics and eggshell mechanical properties. Differences were considered significant at *P* < 0.05. All statistical analyses were performed using Origin Pro (OriginLab Corporation, MA) software package.

## RESULTS AND DISCUSSION

### Egg Properties

Egg properties evaluated in 420 eggs laid by hens from different age groups (33, 45, and 67 wk [n = 140 per group]) are summarized in Figure 1 and Table 1. Eggshell quality parameters (i.e., eggshell breaking strength, eggshell thickness, and eggshell weight percentage) show a gradual decrease with hen age during the production cycle. Still, the registered values for these parameters are generally high for this flock at the end of lay (67 wk). Eggshell breaking strength decreases from 5.8 Kg at 33 wk to 4.4 Kg at 67 wk (25% reduction). Concomitantly, there is also a slight though significant decrease in the eggshell thickness (from 387 µm at 33 wk to 363 µm at 67 wk; 6% reduction) and shell weight percentage (from 12.3% at 33 wk to 11.1% at 67 wk; 10% reduction). The marked decrease of eggshell quality (eggshell thickness, breaking strength) observed in old hens is due in part to a reduction of calcium absorption by the intestine and to an increase in egg size with hen age that the industry is trying to reduce (Nys, 2017). In this study the amount of shell mineral deposited is kept nearly constant (about 7.5 g) during the whole production cycle since the amount of calcium in the diet was increased (from 36 to 41 g/kg) in the second part of the cycle (from 45 to 68 wk) to compensate for the reduced capacity to absorb calcium in older hens. Thus, in this case, the increase in egg size (from 61 g at 33 wk to 67 g at 67 wk) is responsible for the observed reduction in shell weight percentage and eggshell thickness. On the other hand, the amount of eggshell organic matter (eggshell membranes + organic matrix) determined by thermogravimetry was about 4.0% in total, 3% corresponding to the eggshell membranes and the remaining 1% to the organic matter occluded in the shell mineral (eggshell organic matrix). These values stay relatively constant and did not change significantly with hen age.

**Table 1:** Summary of the evolution of main eggshell and egg physical parameters as a function of hen age determined by egg quality analyzer, optical microscopy, X-ray diffraction, and infrared spectroscopy.

	33 wk		45 wk		67 wk			
	Mean	n	Mean	n	Mean	n	F	*P*
Egg weight (g)	60.7 ± 3.3^a^	140	65.3 ± 0.9^b^	140	66.9 ± 4.1^c^	140	96.8	0.000
Breaking strength (Kg)	5.824 ± 0.651^a^	139	5.311 ± 0.909^b^	140	4.391 ± 0.853^c^	140	111.25	0.000
Shell thickness (mm)	0.387 ± 0.018^a^	140	0.372 ± 0.017^b^	140	0.363 ± 0.022^c^	140	53.5	0.000
Shell weight (%)	12.35 ± 0.56^a^	140	11.53 ± 0.54^b^	140	11.07 ± 0.65^c^	140	168.4	0.000
Mammilary_density (mm-1)	21.42 ± 1.64^a^	14	15.01 ± 2.48^b^	14	12.4 ± 0.96^c^	14	92.4	0.000
Organic matter (%)	3.99 ± 0.65^a^	140	4.01 ± 0.73^a^	140	5.72 ± 5.09^b^	140	15.2	0.000
Number of spots	436.7 ± 50.4^a^	140	444.4 ± 38.9^a^	110	253.5 ± 82.5^b^	140	412.0	0.000
Spot intensity (counts)	32909 ± 4361^a^	140	32942 ± 4204^b^	110	75003 ± 31236^c^	140	218.4	0.000
Ratio_110_104	0.219 ± 0.085^a,b^	140	0.255 ± 0.278^a^	139	0.184 ± 0.082^b^	135	5.5	0.004
OH + Amide A	0.227 ± 0.071^a^	140	0.155 ± 0.085^b^	140	0.229 ± 0.087^a^	140	37.6	0.000
Amide I	0.045 ± 0.03^s^	140	0.023 ± 0.022^b^	140	0.039 ± 0.039^s^	140	18.0	0.000
Amide II	0.086 ± 0.015^a^	140	0.073 ± 0.016^b^	140	0.082 ± 0.024^a^	140	18.9	0.000
Carbonates	0.350 ± 0.043 ^a^	140	0.388 ± 0.04 ^b^	140	0.360 ± 0.05^a^	140	27.9	0.000
Phosphate + Sugars	0.104 ± 0.056 ^a^	140	0.061 ± 0.055 ^b^	140	0.087 ± 0.056 ^c^	140	21.0	0.000
Total Cuticle	0.237 ± 0.088 ^a^	140	0.158 ± 0.078 ^b^	140	0.21 ± 0.094 ^c^	140	29.3	0.000
Yolk color factor	11.3 ± 0.6 ^a^	140	11.8 ± 0.5 ^b^	140	10.6 ± 0.6 ^c^	140	147.5	0.000
Albumen height (HU)	80.1 ± 5.1 ^a^	140	78.2 ± 4.8 ^b^	140	69.9 ± 6.9 ^c^	140	123.3	0.000
Yolk index	0.43 ± 0.03 ^a^	140	0.42 ± 0.03 ^b^	140	0.40 ± 0.03 ^c^	140	65.4	0.000

One-way ANOVA was used to test the effect of age on each parameter.

Values of *P* < 0.05 indicate a significant effect of age.

Different lowercase letters in rows represent statistically significant differences in the comparison between weeks (Tukey test).

Additionally, we observed a decrease in the amount of cuticle with hen age in agreement with previous work (Roberts et al., 2013). The majority of eggs that were analyzed have some cuticle deposited on their surface though its distribution over the eggshell surface was highly variable and has an irregular patchy coverage. The analysis of the eggshell outer surface by ATR-FTIR shows that there are notable changes in the signal of the main chemical components of the cuticle (e.g., proteins, phosphate and polysaccharides), or the shell mineral (carbonate), with hen age (see Table 1). In particular, older hens showed lower signal for amides and phosphates+ sugars or total cuticle which indicate that the amount of cuticle deposited on eggs was greater in younger (33 wk) than for older hens (67 wk). A lesser amount of cuticle should have a negative impact in egg safety and quality, as this organic coating is an effective barrier against bacterial penetration (Bain et al., 2013).

There are also notable changes in parameters related to egg internal quality (egg albumen height, YI, yolk color) with hen age (Table 1). Egg albumen height decreases from 88 HU at 33 wk to 70 HU at 67 wk). There is also a gradual decrease in YI from 0.44 at 33 wk to 0.40 at 67 wk. Yolk color fan (**YCF**) slightly increases from 11.3 at 33 wk to 11.7 at 45 wk and decrease 10.6 at 67 wk. All in all, these results indicate that though there is a general decline of egg internal quality with hen age, these parameters are still good at the end of lay (67 wk).

### Eggshell Ultrastructure, Microstructure

The ultra- and microstructure characteristics of the shell of eggs from different hen age groups were studied in detail by optical, electron microscopy, and 2D X-ray diffraction (Figure 1). Although all eggshells have quite similar structural characteristics, there are well-defined variations in the ultra and micro-structure of eggshell with hen age. In particular, there is a large reduction in mammillary density with hen age (from 21.4 mm^−1^ at 33 wk to 12.4 mm^−1^ at 67 wk; 40 % reduction), as mammillary knobs increase in size (width) (from about 45 µm at 33 wk to 83 µm at 67 wk) (Figure 1G and Table 1). On the other hand, 2D X-ray diffraction data reveals that there are also important changes in the size of calcite crystal units with hen age that mirrors the changes observed in mammillary density and size of mammillary knobs being these changes particularly large for eggs laid by 67 wk old birds (Figure 1G and 1H and Table 1). Specifically, the number of spots, that is proportional to the number of calcite crystal units illuminated by the X-ray beam (0.5 mm in diameter), decreases with hen age from about 400 at 33 wk down to 250 at 67 wk, which means that the width of columnar crystal units has significantly increased with hen age mirroring changes in mammillary density (Table 1 and Figure 1G). The increase in size of crystal units is also shown by the increase in spot intensities, a parameter which is directly proportional to crystal size (volume; Figure 1H). On the other hand, there are small changes in the degree of preferential orientation of calcite crystals (I110/I104 ratio), which is low anyway as these eggshells have a nearly random orientation of crystals. These changes with hen age in the eggshell ultra- and microstructure characteristics (i.e., decreased mammillary density; increase in size of crystal units) are in agreement with previous studies (Rodriguez-Navarro et al., 2002; Roberts et al., 2013; Park and Sohn, 2018). These changes should negatively impact eggshell mechanical properties and could explain the marked decrease of eggshell breaking strength with hen age (25%) that cannot be solely explained by the modest reduction of eggshell thickness (only 6 %). In fact, even though, there is well defined correlation between eggshell breaking strength and eggshell thickness (R = 0.454; *P* < 0.001), or eggshell weight percentage (R = 0.640; *P* < 0.001), the latter parameters alone can only explain up to 40% of the variability in breaking strength. On one hand, a decrease in mammillary density reduces the attachment points of the eggshell mineral to the membranes and therefore should negatively impact eggshell mechanical properties. In fact, there is a positive correlation between breaking strength and mammillary density (R = 0.614; *P* < 0.001). On the other hand, the increase of the size of crystal units detected by X-ray diffraction, particularly in the 67 wk old hens’ group, should also negatively impact eggshell mechanical properties. Considering that the eggshell is a ceramic material made of brittle calcite crystal units, though reinforced by the occluded organic matter, an increase in crystal size should negatively impact its mechanical properties, as crack propagation is easier through coarse grain materials (Rodriguez-Navarro et al., 2002). All in all, these results clearly indicate that not only the shell thickness but the ultra- and microstructure characteristics of the shell (mammillary density, crystal size) have an important contribution to eggshell mechanical properties.

Many relevant egg characteristics are known to have an important genetic component and are used in genetically assisted selection programs aimed at improving eggshell quality and safety of eggs (Dunn et al., 2009; Bain et al., 2016). Also, adequate hen nutrition throughout the laying period is very important in order to maintain hen health and improve eggshell quality (Nys, 2017). To compensate the decreased capacity of calcium absorption by the intestine in older hens, higher calcium in diet is used to improve eggshell quality (i.e., eggshell thickness, breaking strength) in older hens. Also, supplying calcium in large particulate form (2–5 mm) that remains available longer in the gut, significantly improving eggshell quality and reduce the mobilization of calcium from the skeleton (Nys, 2017). Additionally, supplying adequate levels of trace metals (Mn, Zn, and Cu), needed for the formation of eggshell and its components, have been shown to significantly improve the quality of eggshell in older hens (Nys, 2017). Interestingly, dietary Mn significantly improved eggshell quality (breaking strength) by modifying the eggshell ultrastructure (increasing mammillary density) (Zhang et al., 2017). The latter is related to Mn enhancing the synthesis of proteoglycans for mammillary knobs formation on the eggshell membrane. This nutritional strategy could be used to correct the reduction in mammillary density observed in older hens and that is probably responsible for the observed marked decrease in eggshell breaking strength in older hens. On the other hand, other strategies can be used to improve eggshell quality in older hens such as feather moulting that has shown to restore eggshell quality temporarily in older hens (Nys, 2017). Moulting induces the renovation of the epithelium of relevant organs (uterus, intestines) increasing calcium absorption capacity and improving eggshell quality.

In summary, this work illustrates the changes occurring in relevant eggshell quality parameters with hen age in a normal production cycle (up to 67 wk). Particularly, changes in eggshell ultra- and microstructure characteristics (i.e., decreased mammillary density; increased size of crystal units) could explain the marked decrease in eggshell breaking strength that cannot be solely explained by the small reduction in eggshell thickness observed. Additionally, there was a decrease in the amount of cuticle with hen age that could have a negative impact in egg safety and quality. Nevertheless, egg external and internal quality parameters were high and still good for this flock at the end of the production cycle (at 67 wk).

## DISCLOSURES

All sources of funding are acknowledged in the manuscript, and authors have declared any direct financial benefits or conflict of interest that could result from publication. All appropriate ethics and other approvals were obtained for the research.
